# Creatine kinase B promotes non–small cell lung cancer survival and metastasis

**DOI:** 10.1016/j.jbc.2025.110805

**Published:** 2025-10-08

**Authors:** Mouna Tlili, Bozena Samborska, Charlotte Girondel, Afnan Abu-Thuraia, Qiaoqiao Zhang, Jakub Bunk, Mohammed F. Hussain, Peter M. Siegel, Lawrence Kazak

**Affiliations:** 1 Rosalind & Morris Goodman Cancer Institute, McGill University, Montreal, Quebec, Canada; 2 Department of Biochemistry, McGill University, Montreal, Quebec, Canada; 3 Department of Medicine, McGill University, Montreal, Quebec, Canada

**Keywords:** creatine, lung cancer, metastasis, osteosarcoma, ovarian cancer

## Abstract

Creatine kinase (CK) catalyzes the reversible transfer of a phosphoryl group from ATP to creatine. There are four distinct CK genes (*CKM* [muscle-type CK], *CKB* [brain-type CK], *CKMT1* [ubiquitous-type CK], and *CKMT2* [sarcomeric-type CK]) with cell-type selective expression and subcellular localization. In cancer, uncontrolled cell proliferation drives aggressive migration and invasion into nearby tissues and distant organs. While creatine metabolism is known to support cancer cell survival, the specific roles of individual CK isoenzymes remain unclear. Here, we demonstrate that CKB is essential for CK enzymatic activity in several cancer cell lines, including non–small cell lung cancer (H1299), osteosarcoma (143B), and ovarian adenocarcinoma (OVCAR8). Moreover, we demonstrate that CKB promotes metastasis of H1299 cells to the lung and liver *in vivo*, a process associated with enhanced anoikis resistance.

Creatine kinase (CK) catalyzes the reversible transfer of a phosphoryl group from ATP to creatine, playing a key role in cellular energy homeostasis (1, 2). The four CK isoenzymes, *CKM* (muscle-type CK), *CKB* (brain-type CK), *CKMT1* (ubiquitous-type CK), and *CKMT2* (sarcomeric-type CK), are compartmentalized within cells at sites of ATP production and utilization. Their activity regulates the balance between ATP supply and demand (3). Aberrant CK expression has been linked to poor prognosis in various cancers (4, 5, 6, 7, 8, 9, 10, 11, 12, 13, 14, 15, 16, 17, 18, 19). Genetic and pharmacological approaches have demonstrated that reducing CK activity may have therapeutic potential at reducing tumor burden, limiting metastasis, and improving survival (10, 11, 15, 16, 20, 21, 22, 23, 24). CK is a poor prognostic factor in lung cancer (25, 26, 27); however, the specific CK isoenzyme and the requirement for CK in lung metastasis are not fully understood.

In this study, we show that CK expression levels correlate with sensitivity to cyclocreatine, a synthetic creatine analog that competes with native CK metabolites, reducing the phosphoryl transfer capacity within cells (16, 28). We show that CKB is required for non–small cell lung cancer (NSCLC) metastasis to the lung and liver *in vivo*, not by altering migration or chemotaxis, but rather by promoting resistance to detachment-induced cell death (anoikis).

## Results

### Selective reduction in cell survival by the CK inhibitor, cyclocreatine

**Figure 1:**
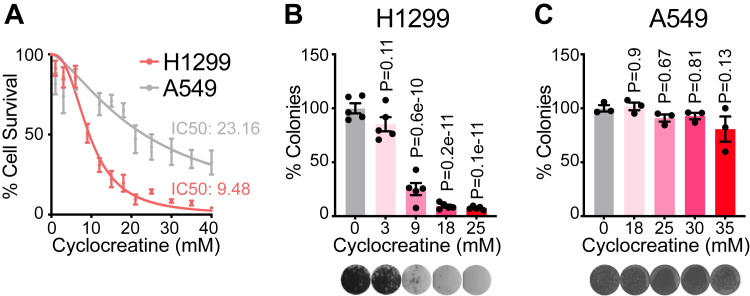
**Selective reduction in cell survival by the creatine kinase inhibitor, cyclocreatine.***A*, cell survival analysis, as determined by SRB staining, of H1299 (*n* = 10) and A549 (*n* = 7) cells treated for 3 days with cyclocreatine. *B* and *C*, colony formation assays of H1299 (*n* = 5) and A549 (*n* = 3) cells. Crystal violet staining was analyzed by ImageJ software. Data are presented as mean ± SEM. *n* numbers are of biologically independent experiments. *B* and *C*, one-way ANOVA (Dunnett’s multiple comparison test). SRB, Sulforhodamine B.

To investigate the role of CK in NSCLC progression, we examined two distinct NSCLC cell lines, H1299 and A549, which exhibit notable differences in gene expression, drug sensitivity, and metastatic potential (29, 30). First, we assessed their sensitivity to CK inhibition using cyclocreatine. Cells were treated with increasing concentrations of cyclocreatine, and after 72 h, cell viability was analyzed. H1299 cells displayed an IC_50_ of 9.48 mM, whereas A549 cells exhibited a significantly higher IC_50_ of 23.16 mM (Fig. 1*A*). Thus, H1299 cells are more sensitive to cyclocreatine than A549 cells, suggesting a differential reliance on CK activity. Next, we performed a colony formation assay to assess the ability of single cells to proliferate into colonies in the presence of cyclocreatine. H1299 cells exhibited a significant, dose-dependent reduction in colony formation (Fig. 1*B*), whereas A549 cells could tolerate a much higher cyclocreatine concentration in comparison (Fig. 1*C*). These data indicate that CK inhibition effectively suppresses H1299 cell expansion but has minimal impact on A549 growth, prompting us to explore the underlying mechanisms driving these differences.

### CK activity in H1299 cells is mediated by CKB

**Figure 2:**
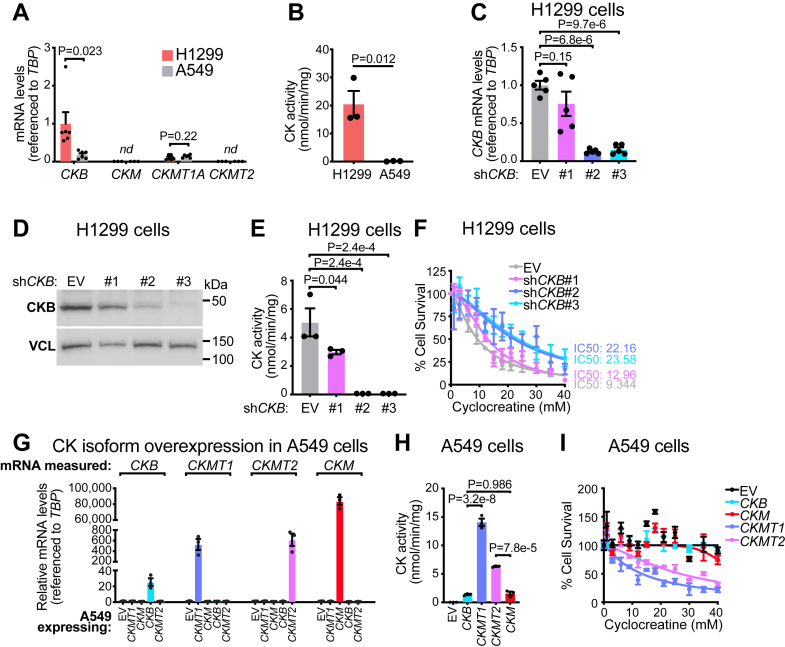
**Creatine kinase (CK) activity in H1299 cells is mediated by brain-type CK (CKB).***A*, relative mRNA expression of *CKB*, *CKM*, *CKMT1*, and *CKMT2* in H1299 (*n* = 6, 3, 6, and 3) and A549 (*n* = 6, 3, 6, and 3). *B*, CK activity in protein lysates from H1299 and A549 cells (*n* = 3 per group). *C*, relative *CKB* mRNA expression in H11299 cells stably expressing empty vector (EV), sh*CKB*#1, sh*CKB*#2, or sh*CKB*#3 (*n* = 5 per group). *D*, Western blot of H1299 cells stably expressing EV, sh*CKB*#1, sh*CKB*#2, or sh*CKB*#3. *E*, CK activity in protein lysates from H1299 cells stably expressing EV, sh*CKB*#1, sh*CKB*#2, or sh*CKB*#3 (*n* = 3 per group). *F*, cell viability analysis, as determined by SRB staining, was treated for 3 days with cyclocreatine (*n* = 6, 4, 5, 5 for EV, sh*CKB*#1, sh*CKB*#2, sh*CKB*#3, respectively). *G*, relative *CK* isoform mRNA expression in A549 cells stably expressing EV, *CKB*, *CKMT1*, *CKMT2*, or *CKM* (*n* = 3 per group). *H*, CK activity in protein lysates from A549 cells stably expressing EV, *CKB*, *CKMT1*, *CKMT2*, or *CKM* (*n* = 3 per group). *I*, cell viability analysis, as determined by SRB staining, treated for 3 days with cyclocreatine (*n* = 3 per group). Data are presented as mean ± SEM. *n* numbers are of biologically independent experiments. *A*, two-way ANOVA (Šidák’s multiple comparison test); (*B*) two-tailed Student’s *t* test; (*C* and *E*) one-way ANOVA (Dunnett’s multiple comparison test); (*H*) one-way ANOVA (Tukey’s multiple comparison test). *CKB*, brain-type CK; *CKM*, muscle-type CK; *CKMT1*, ubiquitous-type CK; *CKMT2*, sarcomeric-type CK; SRB, Sulforhodamine B.

To determine whether the sensitivity of H1299 cells to cyclocreatine correlates with elevated CK expression and to identify the specific CK isoenzyme responsible, we analyzed the mRNA levels of the four CK isoforms: *CKM*, *CKB*, and the mitochondrial isoforms *CKMT1* and *CKMT2*. Gene expression analysis revealed that *CKB* levels were significantly higher in H1299 cells compared with A549 cells (Fig. 2*A*), suggesting a key role for this isoenzyme in mediating CK activity and cyclocreatine sensitivity. In contrast, the expression of *CKM*, *CKMT1*, and *CKMT2* did not differ between the two cell lines (Fig. 2*A*), indicating that CKB is the primary CK isoenzyme contributing to the observed differences in cyclocreatine response. Furthermore, H1299 cells displayed strong CK activity, whereas A549 cells exhibited negligible CK activity (Fig. 2*B*). These findings indicate that high CK activity in H1299 cells underlies their sensitivity to cyclocreatine, whereas the near lack of CK activity in A549 cells accounts for their resistance.

To assess the contribution of CKB to total CK activity, we generated a *CKB* loss-of-function model in H1299 cells using shRNA-mediated gene knockdown (KD). The three shRNAs targeting *CKB* exhibited varying KD efficiencies. While sh*CKB*#1 reduced *CKB* mRNA expression by 20%, sh*CKB*#2 and sh*CKB*#3 achieved more substantial silencing (∼90% reduction) (Fig. 2*C*). Similar KD efficiencies were observed at the protein level (Fig. 2*D*). CK activity assays revealed that KD of *CKB* strongly correlated with a reduction in CK activity: CK activity was abolished in sh*CKB*#2 and sh*CKB*#3 cells and partially reduced in sh*CKB*#1 cells (Fig. 2*E*). We next examined whether *CKB* KD cells lose their sensitivity to cyclocreatine. In control cells, the IC_50_ was 9.34 mM, whereas *CKB* KD cells (sh*CKB*#1, sh*CKB*#2, and sh*CKB*#3) exhibited significantly higher IC_50_ values of 12.96 mM, 22.16 mM, and 23.58 mM, respectively (Fig. 2*F*). This indicates that *CKB* silencing induces resistance to cyclocreatine treatment in H1299 cells. Importantly, we found no change in *CKMT1* levels in *CKB*-silenced H1299 cells (Fig. S1*A*), suggesting that *CKMT1* did not contribute to the phenotype upon *CKB* reduction. Moreover, when we generated H1299 cells stably expressing three independent shRNAs targeting *CKMT1*, we could only reduce *CKMT1* levels by a maximum of ∼20% (Fig. S1*B*), which is consistent with low basal *CKMT1* expression. Moreover, H1299 cells expressing shRNAs targeting *CKMT1* exhibited unaltered CK activity and maintained cyclocreatine sensitivity (Fig. S1, *C* and *D*). Together, these findings demonstrate that CK activity and cyclocreatine sensitivity is tightly linked with *CKB* expression, strongly indicating that CKB is the primary determinant of CK activity in H1299 cells.

We next tested whether genetically overexpressing CK in A549 cells could modulate their cyclocreatine sensitivity. We generated stable cells expressing *CKMT1*, *CKMT2*, *CKM*, or *CKB*, which showed clear overexpression of the intended isoenzyme, confirming successful expression (Fig. 2*G*). CK overexpression also restored functional CK activity (Fig. 2*H*), with the mitochondrial-exclusive isoenzymes exhibiting the highest activity, disproportionate to their relative mRNA levels. This is consistent with a major kinetic distinction, where mitochondrial forms have a higher affinity for ADP than cytosolic forms (31, 32). Despite *CKM* mRNA levels being ∼10-fold higher than *CKMT2*, *CKMT2*-expressing lysates displayed ∼4-fold greater activity (Figure 2, *G* and *H*). CKMT1 activity exceeded CKB because of higher expression and smaller kinetic differences between these isoenzymes, whereas *CKB* mRNA was much lower than *CKM*, yet yielded similar activity, indicating intrinsically higher CK activity in *CKB*-expressing cell lysates, consistent with published reports (33, 34). We examined how overexpressing CK isoenzymes in CK-deficient cells influenced cyclocreatine sensitivity. *CKMT1* and *CKMT2* conferred cyclocreatine sensitivity to A549 cells, whereas *CKB* and *CKM* showed minimal effect (Fig. 2*I*). These findings indicate that sensitivity is dictated either by overall catalytic activity, isoenzyme identity, or localization. These possibilities are also not mutually exclusive, and the combination of these factors could amplify the response.

### CKB silencing does not affect cell viability or ATP levels

**Figure 3:**
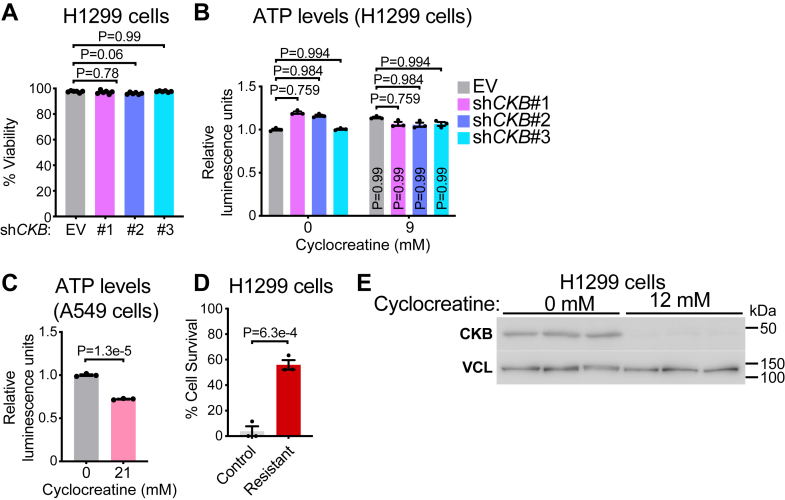
**CKB silencing does not affect cell viability or ATP levels.***A*, cell viability of H1299 cells stably expressing empty vector (EV), sh*CKB*#1, sh*CKB*#2, or sh*CKB*#3 (*n* = 6 per group). *B*, ATP levels in H1299 cells stably expressing EV, sh*CKB*#1, sh*CKB*#2, or sh*CKB*#3 and treated with vehicle or 9 mM cyclocreatine for 24 h (*n* = 3 per group). Data are expressed relative to 0 mM cyclocreatine treatment of EV-expressing cells and normalized by cell number. *p* Values in the bar graphs represent comparisons of cyclocreatine treatment within each cell type (EV, sh*CKB*#1, sh*CKB*#2, or sh*CKB*#3). *C*, ATP levels in A549 cells treated with vehicle or 21 mM cyclocreatine for 24 h (*n* = 3 per group). Data are expressed relative to 0 mM cyclocreatine and normalized by cell number. *D*, Cell viability analysis, as determined by SRB staining, treated for 3 days with 40 mM cyclocreatine (*n* = 3 per group). *E*, Western blot of control and cyclocreatine-resistant H1299 cells. Data are presented as mean ± SEM. *n* numbers are of biologically independent experiments. *A*, one-way ANOVA (Dunnett’s multiple comparison test). *B*, two-way ANOVA (Šidák’s multiple comparison test). *C* and *D*, two-tailed Student’s *t* test. *CKB*, brain-type CK; SRB, Sulforhodamine B.

To assess whether CKB KD impairs general cell health, we measured cell viability by trypan blue staining and found no difference between control and CKB-silenced H1299 cells (Fig. 3*A*). We next asked whether the reduction in H1299 cell number following cyclocreatine treatment could be explained by ATP depletion. However, ATP levels in H1299 cells were not significantly affected by cyclocreatine (Fig. 3*B*). In contrast, A549 cells, which are resistant to cyclocreatine-induced growth suppression, exhibited a greater reduction in ATP levels after 24 h of cyclocreatine treatment (Fig. 3*C*). These findings indicate that the antiproliferative effects of cyclocreatine in H1299 cells are not because of general toxicity or ATP depletion but rather disruption of CKB-dependent processes critical for cell expansion. This is consistent with data showing that cyclocreatine inhibits tumor growth *in vivo* without depleting ATP (23). The disconnect between ATP depletion and resistance to cyclocreatine-mediated growth suppression in A549 cells further supports this interpretation. It remains possible that bioenergetic function is preserved even with reduced ATP levels if the free energy of hydrolysis is maintained, a possibility that warrants further study. However, such changes are unlikely to be detectable at the whole-cell level. Moreover, cells with compromised energetics might be cleared, complicating efforts to causally link adenylate levels to cell number.

Next, we asked whether A549 cell resistance to cyclocreatine is intrinsic or reflects metabolic reprogramming that occurs in the absence of CK activity. To test this, we cultured H1299 cells under gradually increasing concentrations of cyclocreatine, up to 12 mM (above their IC_50_), and observed that they acquired resistance. Notably, these cells also remained resistant when treated at a high concentration of 40 mM cyclocreatine (Fig. 3*D*). This adaptation was accompanied by a marked reduction in CKB protein levels (Fig. 3*E*), independently validating the central role of CKB in supporting growth. Such downregulation is a well-established mechanism of acquired resistance, as seen with reduced folate carrier expression or impaired polyglutamylation in methotrexate resistance (35), loss of estrogen receptor levels in tamoxifen-treated cancers (36), and decreased epidermal growth factor receptor expression following epidermal growth factor receptor inhibitor therapy (37). These findings suggest that, over time, cancer cells engage alternative metabolic pathways to bypass reliance on CK activity, a possibility that merits future investigation.

### CKB mediates anoikis resistance *in vitro*

**Figure 4:**
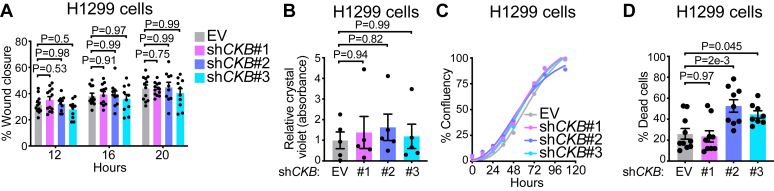
**CKB mediates anoikis resistance *in vitro*.***A*, percent wound closure of H1299 cells stably expressing EV, sh*CKB*#1, sh*CKB*#2, or sh*CKB*#3 (*n* = 12, 12, 10, and 10) 12 h, 16 h, and 20 h following scratch. *B*, cell migration of H1299 cells stably expressing EV, sh*CKB*#1, sh*CKB*#2, or sh*CKB*#3 (*n* = 6, 4, 5, and 5). *C*, percent confluency of H1299 cells stably expressing EV, sh*CKB*#1, sh*CKB*#2, or sh*CKB*#3 (*n* = 3 per group). *D*, anoikis of H1299 cells stably expressing EV, sh*CKB*#1, sh*CKB*#2, or sh*CKB*#3 (*n* = 10, 9, 9, and 8). Data are presented as mean ± SEM. *n* numbers are of biologically independent experiments. *A*, two-way ANOVA (Dunnett’s multiple comparison test). *B* and *D*, one-way ANOVA (Dunnett’s multiple comparison test). *CKB*, brain-type CK; EV, empty vector.

Our experiments revealed that inhibiting CK activity in H1299 cells with cyclocreatine reduced cell survival, an effect mediated primarily through inhibition of CKB function. To further explore the role of CKB in NSCLC progression, we investigated its role in metastasis. We first assessed cell migration, a key step in tumor invasion (38, 39), using a wound-healing assay. *CKB*-silenced H1299 cells displayed similar wound closure rates as control cells (Fig. 4*A*), suggesting that cell–cell interactions and motility remain intact. In addition, we evaluated chemotactic migration, finding no difference between *CKB*-silenced and control cells in their ability to migrate toward a chemoattractant gradient (Fig. 4*B*). These findings indicate that CKB does not influence H1299 cell migration. To rule out proliferation defects as a confounding factor, we monitored cell proliferation over 5 days and observed no differences between *CKB* KD and control cells (Fig. 4*C*). Thus, *CKB* silencing neither impairs migration nor is it linked to proliferation deficits, at least under nutrient-replete conditions *in vitro*.

Metastasis requires multiple steps, including detachment from the primary tumor, survival in circulation, and colonization of distant organs (38, 39). Since *CKB-*silenced cells do not show a defect in migration, we next investigated the process of intravasation, where cancer cells must resist anoikis (40). When cultured under low-attachment conditions for 5 to 7 days, *CKB*-silenced cells (sh*CKB*#2 and sh*CKB*#3) showed significantly higher levels of cell death compared with control cells (Fig. 4*D*), suggesting that CKB helps cells resist anoikis. In contrast, cells expressing sh*CKB*#1, which only weakly reduced CKB levels (Fig. 2*C*), did not show increased cell death (Fig. 4*D*). Thus, while *CKB* KD does not affect migration, it compromises anoikis resistance *in vitro*, suggesting a potential reduction in H1299 metastatic capacity.

### CKB promotes metastasis *in vivo*

**Figure 5:**
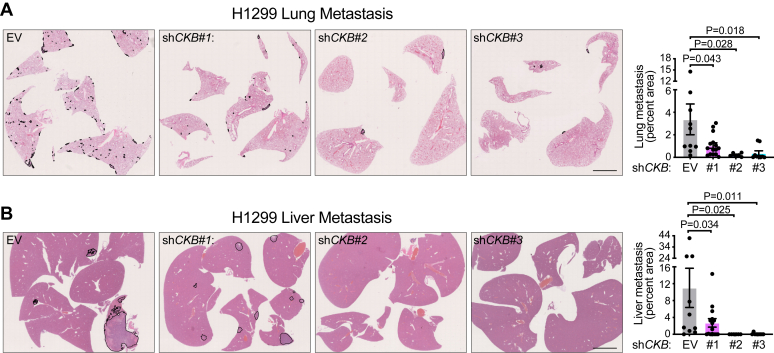
**CKB promotes metastasis *in vivo*.***A*, representative H&E-stained images of lung metastasis in NSG mice and their quantification (percent area) following intravenous injection of H1299 cells stably expressing EV, sh*CKB*#1, sh*CKB*#2, or sh*CKB*#3 (*n* = 10, 15, 6, and 9). *B*, representative H&E-stained images of liver metastasis in NSG mice and their quantification (percent area) following intravenous injection of H1299 cells stably expressing EV, sh*CKB*#1, sh*CKB*#2, or sh*CKB*#3 (*n* = 10, 15, 6, and 9). Data are presented as mean ± SEM. n numbers are of biologically independent experiments. *A* and *B*, one-way ANOVA (Dunnett’s multiple comparison test). *CKB*, brain-type CK; EV, empty vector.

Metastasis is a complex process involving cell migration, invasion, intravasation into the bloodstream or lymphatic system, and colonization at distant sites (38, 39, 41, 42). Normally, cells that detach from the extracellular matrix undergo anoikis to prevent uncontrolled dissemination (43). However, malignant cells develop mechanisms to resist anoikis, allowing them to survive in circulation and seed secondary tumors (40, 44). Metabolic reprogramming is a key driver of anoikis resistance, yet the role of CK in this process, particularly in NSCLC, remains poorly understood (45). To investigate the role of CKB in metastasis, we used an experimental metastasis model in which CKB-expressing and CKB-silenced H1299 cells were injected into the lateral tail vein. NSG mice were divided into four groups: a control group (empty vector [EV]) and three CKB KD groups (sh*CKB*#1, sh*CKB*#2, and sh*CKB*#3). After 5 weeks, mice were euthanized, and their lungs and livers were collected, formalin fixed, and paraffin embedded. Upon examination of H&E sections, the control group exhibited numerous metastatic nodules in both the lungs (Fig. 5*A*) and liver (Fig. 5*B*), whereas *CKB*-silenced groups showed significantly fewer nodules. Representative H&E images confirmed that metastatic nodules in control mice had large, irregular nuclei in both the lungs and liver (Fig. 5, *A* and *B*). In contrast, sh*CKB*#1 mice exhibited fewer and smaller nodules, whereas metastases were barely detectable in sh*CKB*#2 and sh*CKB*#3 groups, reflecting the degree of *CKB* silencing (Fig. 2*C*). Quantification of metastatic burden across both lungs and all liver lobes revealed a significantly reduced metastatic area in *CKB*-silenced groups, with the strongest effect in sh*CKB*#2 and sh*CKB*#3. These findings suggest that CKB expression is a key regulator of H1299 metastasis. Our results demonstrate that *CKB* silencing reduces tumor progression by limiting metastasis to the lungs and liver, highlighting CKB as a potential therapeutic target for NSCLC metastatic lung cancer.

### CKB is the predominant active isoenzyme in bone and ovarian cancer

**Figure 6:**
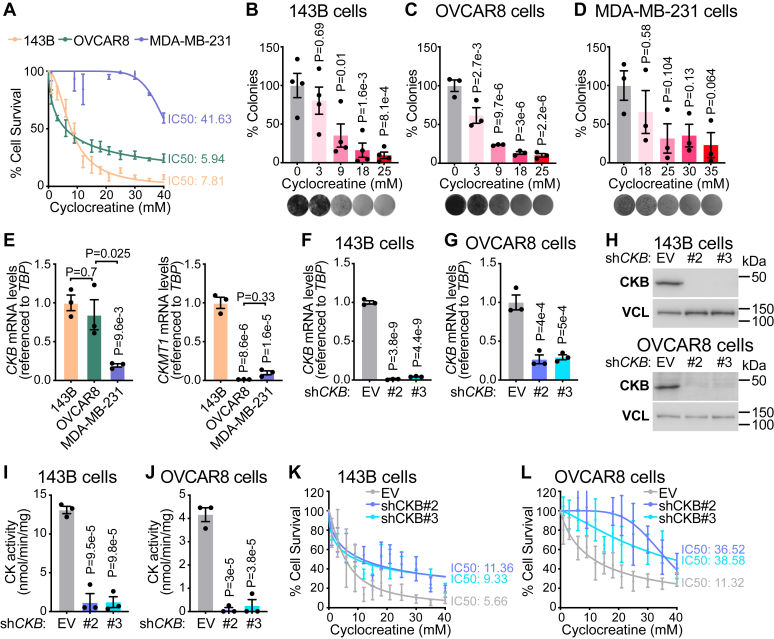
**CKB is the predominant active isoenzyme in bone and ovarian cancer.***A*, cell survival analysis, as determined by SRB staining, of 143B (*n* = 3–6 per dose), OVCAR8 (*n* = 4–5 per dose), and MDA-MB-231 (*n* = 3–6 per dose) cells treated for 3 days with cyclocreatine. *B*–*D*, colony formation assays of 143B (*n* = 4 per group), OVCAR8 (*n* = 3 per group), and MDA-MB-231 (*n* = 3 per group) cells. Crystal violet staining was analyzed by ImageJ software. *E*, relative *CKB* and *CKMT1* mRNA expression in 143B, OVCAR8, and MDA-MB-231 cells (*n* = 3 per group). *F*, relative *CKB* mRNA expression in 143B cells stably expressing empty vector (EV), sh*CKB*#2, or sh*CKB*#3 (*n* = 3 per group). *G*, relative *CKB* mRNA expression in OVCAR8 cells stably expressing EV, sh*CKB*#2, or sh*CKB*#3 (*n* = 3 per group). *H*, Western blot of 143B and OVCAR8 cells stably expressing EV, sh*CKB*#2, or sh*CKB*#3. *I*, CK activity in protein lysates from 143B cells stably expressing EV, sh*CKB*#2, or sh*CKB*#3 (*n* = 3 per group). *J*, CK activity in protein lysates from OVCAR8 cells stably expressing EV, sh*CKB*#2, or sh*CKB*#3 (*n* = 3 per group). *K*, cell survival analysis, as determined by SRB staining, of 143B cells treated for 3 days with cyclocreatine (*n* = 4 per group). *L*, cell survival analysis, as determined by SRB staining, of OVCAR8 cells treated for 3 days with cyclocreatine (*n* = 4 per group, except for *n* = 3 for sh*CKB*#2 and sh*CKB*#3 at 9 mM and 25 mM). Data are presented as mean ± SEM. *n* numbers are of biologically independent experiments. *B*–*G*, *I* and *J*, one-way ANOVA (Dunnett’s multiple comparison test). CK, creatine kinase; *CKB*, brain-type CK; SRB, Sulforhodamine B.

Thus far, our findings have established CKB as a key regulator of metastasis in H1299 cells, a subtype of NSCLC. To determine whether CKs play a similar role in other cancers, we examined metastatic cell lines from bone (143B), ovarian (OVCAR8), and breast cancer (MDA-MB-231) (46, 47, 48). We first assessed the response of these cell lines to cyclocreatine. 143B, OVCAR8, and MDA-MB-231 cells showed an IC_50_ of 7.81 mM, 5.94 mM, and 41.63 mM, respectively (Fig. 6*A*), indicating higher sensitivity in bone and ovarian cancer cells. This trend was further validated using colony formation assays, reinforcing the hypothesis that CK activity differs across these cell lines (Fig. 6, *B*–*D*). To determine which CK isoenzymes are responsible for this sensitivity, we first analyzed mRNA expression of key CK variants. The cyclocreatine-sensitive cell lines (143B and OVCAR8) expressed higher levels of *CKB* than the cyclocreatine-resistant MDA-MB-231 cell line (Fig. 6*E*). On the other hand, *CKMT1* was detectable in 143B cells but not OVCAR8 or MDA-MB-231 cells (Fig. 6*E*). *CKMT2* and *CKM* were not expressed in any of the three cell lines (data not shown). These results correlate cyclocreatine sensitivity with *CKB* expression, suggesting that CKB is the predominant functional CK isoenzyme in 143B and OVCAR8 cells. To explore this hypothesis further, we generated 143B and OVCAR8 cells stably expressing shRNAs targeting *CKB* or *CKMT1* (Fig. S2, *A* and *B*). We achieved a significant reduction in *CKB* mRNA in both 143B and OVCAR8 cells with two independent shRNAs (Fig. 6, *F* and *G*), which resulted in a dramatic decrease in CKB protein (Fig. 6*H*) and CK activity (Fig. 6, *I* and *J*). Importantly, *CKMT1* mRNA levels were unchanged in *CKB*-silenced 143B and OVCAR8 cells (Fig. S2, *A* and *B*), indicating that the effects on CK activity were due to *CKB*. Next, we silenced *CKMT1* in 143B cells (Fig. S2*A*), which had no impact on *CKB* mRNA levels (Fig. S2*C*). We could not decrease *CKMT1* mRNA in OVCAR8 cells (Fig. S2*B*), likely because these cells express negligible *CKMT1* levels, and this too had no effect on *CKB* mRNA abundance (Fig. S2*D*). Strikingly, despite a reduction in *CKMT1* mRNA levels in 143B cells (Fig. S2*A*), CK activity was unchanged (Fig. S2*E*), contrasting the results obtained with *CKB* silencing (Fig. 6*I*). As expected, OVCAR8 cells stably expressing *CKMT1*-targeted shRNAs exhibited comparable CK activity to control cells (Fig. S2*F*). Next, we examined how *CKB* silencing affected cyclocreatine sensitivity. In control 143B cells, the IC_50_ was 5.66 mM, whereas *CKB* KD cells (sh*CKB*#2 and sh*CKB*#3) exhibited higher IC_50_ values of 11.36 mM and 9.33 mM, respectively (Fig. 6*K*). Similarly, the IC_50_ in control OVCAR8 cells (11.32 mM) was substantially lower than sh*CKB*#2 (36.52 mM) and sh*CKB*#3 (38.58 mM) cells (Fig. 6*L*). In contrast, cells expressing shRNAs targeting *CKMT1* remained highly sensitive to cyclocreatine (Fig. S2, *G* and *H*), further emphasizing a minimal role for *CKMT1* in these cells. Therefore, we conclude that, like H1299 cells, CKB is the main active CK isoenzyme in 143B and OVCAR8 cells, highlighting CKB as a potential therapeutic target in these malignancies.

## Discussion

Here, we demonstrate that CKB is a key regulator of metastasis in H1299 NSCLC cells. Numerous studies have reported that the expression of various CK isoenzymes is upregulated in cancers, compared with healthy tissues (5, 8, 17, 49). Recognizing this, we sought to determine the expression patterns of different CK isoenzymes in NSCLC. Our findings reveal that CKB is more abundantly expressed than all other CK isoforms at the mRNA level in H1299 cells and that CKB is required for essentially all the CK activities in these cells. Our findings complement prior work that has demonstrated a role for CKB in promoting cell survival, migration, chemotaxis, and metastasis in hepatocellular carcinoma, pancreatic ductal adenocarcinoma, prostate cancer, osteosarcoma, and estrogen receptor–negative breast cancer (6, 11, 50, 51, 52). Our data place NSCLC among the growing list of cancers regulated by CKB. *CKMT1* inactivation was reported to disrupt the cell cycle and reduce tumor growth in A549 cells (17); however, we found that A549 cells were resistant to cyclocreatine treatment and exhibited low CK activity, suggesting a minor role for endogenous CK in these cells. However, enforced overexpression of *CKMT*1 and *CKMT2* was sufficient to render otherwise resistant A549 cells sensitive to cyclocreatine, revealing a chokepoint in A549 cells that relies on mitochondrial CK activity. By contrast, *CKB* and *CKM* overexpression did not confer sensitivity, likely because their total CK activity was lower. Thus, the difference in sensitivity between mitochondrial (CKMT1/2) and cytosolic (CKB/CKM) isoenzymes may reflect overall catalytic capacity or activity within specific microdomains. In H1299 cells, however, endogenous cytosolic CKB is required for cyclocreatine sensitivity. Hence, we conclude that mitochondrial CK is sufficient to drive cyclocreatine sensitivity in A549 cells, whereas H1299 cells rely on endogenous CKB to mediate their sensitivity endogenously.

Clinically, low plasma CK levels have been correlated with lung cancer metastasis and poor survival (27), suggesting that CK may facilitate cancer progression. Consistently, in colon cancer, extracellular CKB sustains cell survival in hypoxic conditions during transit to metastatic sites in the liver (21). Our data show that *CKB* silencing increases anoikis sensitivity in H1299 cells, leading to enhanced cell death in suspension cultures, a condition that mimics circulating tumor cells *in vivo* (53). Furthermore, our *in vivo* experiments reveal a reduction in liver metastases by CKB-depleted H1299 cells, with metastasis rates directly correlating with CKB expression. These findings underscore the critical function of CKB in promoting NSCLC metastasis.

We also extend our analysis to other metastatic cell lines, such as OVCAR8 (high-grade serous ovarian cancer) (47) and 143B (osteosarcoma) (54), both of which we show rely on CKB for CK activity, making them susceptible to CK-targeting therapies. Of course, therapeutic targeting of CKB will need to be tempered against potential side effects mediated by its inhibition in normal cells that require its activity. However, there might be a therapeutic window, particularly when combined with other therapies. In summary, our study identifies CKB as a regulator of NSCLC metastasis, supporting the rationale for pursuing CK-targeted strategies for combating lung cancer progression.

## Experimental procedures

### Cell lines

All cell lines were grown in Dulbecco's modified Eagle's medium (DMEM) (Wisent, Inc; catalog no.: 319-005 CL) supplemented with 10% fetal bovine serum (FBS) (Wisent, Inc; catalog no.: 090-105) and 1% penicillin–streptomycin (P–S; Wisent, Inc; catalog no.: 450-201-EL). Cells were maintained at 37 °C in a humidified 5% CO_2_ environment. Cell line sources: H1299: American Type Culture Collection (ATCC; CRL-5803); A549: ATCC (CCL-185), OVCAR8: Dr Sidong Huang (McGill University), 143B: ATCC (CRL-8303), and MDA-MB-231: ATCC (CRM-HTB-26).

### Lentivirus production and transduction

All experiments with ectopic expression or shRNA KD were performed using lentiviral constructs. For lentivirus production, human embryonic kidney 293T cells were grown in DMEM (Wisent, Inc; catalog no.: 319-005 CL) supplemented with 10% FBS (Wisent, Inc; catalog no.: 090-105) and 1% P–S (Wisent, Inc; catalog no.: 450-201-EL) until reaching 60% to 70% confluency. Cells were washed with 1X PBS (Wisent, Inc; catalog no.: 311-010 CL), and the medium was changed for Opti-MEM I Reduced-Serum Medium (Gibco; catalog no.: 31985062). Cells were transfected with lentiviral constructs, the packaging (psPAX2), envelope plasmid (pMD2.G), and Lipofectamine 2000 (Fisher Scientific; catalog no.: 11668019) as per the manufacturer's recommendations for ∼6 h before changing the medium. Virus-containing medium was harvested at 48 h after transfection, centrifuged at 500*g*, 5 min, filtered through a 0.45 μm syringe filter (Corning; catalog no.: 431220), and stored at −80 °C.

For transduction, ∼85 × 10^3^ H1299 or 143B cells, ∼170 × 10^3^ OVCAR8 cells, and 130 × 10^3^ A549 cells were seeded the day before in 6-well plates to reach 40% confluency the next day. Cells were infected with the virus overnight. After ∼24 to 48 h recovery, cells were selected in medium containing 1 to 2 μg/ml puromycin (Biobasic; catalog no.: PJ593) for 4 days (shRNA KD) or 6 to 7 days (ectopic overexpression). Cells were then expanded in regular medium and harvested for the experiments.

### CK isoform silencing

Individual shRNA vectors used were from the MISSION TRC library provided by the McGill Platform for Cellular Perturbation of Rosalind and Morris Goodman Cancer Institute and Department of Biochemistry at McGill University. sh*CKB*#1 (TRCN0000315324), sh*CKB*#2 (TRCN0000315376), and sh*CKB*#3 (TRCN0000356572); sh*CKMT1*#1 (TRCN0000243704), sh*CKMT1*#2 (TRCN0000243701), and sh*CKMT1*#3 (TRCN0000243700). pLKO—TRC005 control was used as EV control.

### CK isoform overexpression

Individual ORF vectors were from the MISSION TRC library provided by the McGill Platform for Cellular Perturbation of Rosalind and Morris Goodman Cancer Institute and Department of Biochemistry at McGill University: CKMT1a ORF (TRCN0000471987), CKM ORF (TRCN0000468913), CKB ORF (TRCN0000469112), and CKMT2 (TRCN0000480045). pLEX_307 (Addgene #41391) was used as the EV control.

### Colony formation assay

H1299, A549, 143B cells (1000 cells per well of a 6-well dish), and OVCAR8 cells (3000 cells per well of a 6-well dish) were treated with cyclocreatine (2-imino-1-imidazolidineacetic acid, 98%) (Sigma–Aldrich; catalog no.: 37762) for ∼10 days or until the control (untreated plate) reached 90% to 100% confluency. Medium was refreshed twice a week. At the endpoint, cells were fixed with 4% formaldehyde, stained with crystal violet (Sigma–Aldrich; catalog no.: C3886) (0.1% w/v), washed with water, and imaged. Experiments were performed independently at least three times.

### Generation of cyclocreatine-resistant H1299 cells

H1299 cells were gradually adapted to cyclocreatine by stepwise dose escalation, starting at 1 mM and increasing to 3, 6, 9, and 12 mM at 3- to 4-week intervals. Control cells were cultured in parallel without cyclocreatine.

### Sulforhodamine B assay

Six thousand cells were seeded in a 24-well plate. The next day, the cells were treated with 1 ml of the indicated doses of cyclocreatine for 72 h and subsequently fixed at 4 °C for 1 h with 25% acetic acid. Fixed cells were washed three times with 1 ml of water and dried under the chemical hood for ∼20 min. Cells were stained with 0.057% Sulforhodamine B (Sigma–Aldrich; catalog no.: 230162), dissolved in 1% acetic acid, and incubated for 30 min at room temperature, protected from light. Cells were washed three times with 1% acetic acid and completely dried. Sulforhodamine B was solubilized in 500 μl of 10 mM Tris, pH 10, solution. The absorbance was measured at 510 nm.

### Cell viability using trypan blue staining

About 250 × 10^3^ H1299 cells were seeded in 6-well plates. The next day, the cells were mixed 1:1 with 0.4% trypan blue (Gibco; catalog no.: 15250061). Viable and dead cells were counted manually using a hemocytometer.

### ATP levels using Cell Titer Glo

Five thousand H1299 or A549 cells were seeded in 0.2 ml of complete DMEM (Wisent, Inc; catalog no.: 319-005 CL) supplemented with 10% FBS (Wisent, Inc; catalog no.: 090-105) and 1% P–S (Wisent, Inc; catalog no.: 450-201-EL), in a 96-well white wall clear bottom plate (Greiner Bio-One; catalog no.: 655098). The following day, the medium was replaced with either 0.1 ml of complete media or 0.1 ml of complete media containing cyclocreatine at the IC_50_ for each cell line (9 mM for H1299 cells and 21 mM for A549 cells). Twenty-four hours after cyclocreatine addition, ATP was measured using the CellTiter-Glo Luminescent cell viability assay (Promega; G7570), following the manufacturer’s instructions. Luminescence was read from the top of the plate. A parallel plate was used to count cells from each condition. The cell number from each cell line and treatment condition was used to normalize the luminescence values to obtain the reported relative luminescence units.

### Western blotting

Samples were prepared in lysis buffer (50 mM Tris, pH 7.4, 500 mM sodium chloride, 1% NP-40, 20% glycerol, 5 mM EDTA, and 1 mM PMSF), supplemented with a cocktail of Roche protease inhibitors. The homogenates were centrifuged at 16,000*g* for 15 min at 4 °C, and the supernatants were used for subsequent analyses. Protein concentration was determined using the bicinchoninic acid assay (Pierce). Ten micrograms of protein lysate were used for Western blot analysis. Protein lysates were denatured in Laemmli buffer (60 mM Tris, pH 6.8, 2% SDS, 10% glycerol, 0.05% bromophenol blue, and 0.7 M β-mercaptoethanol), resolved by 10% Tris–glycine SDS-PAGE, and transferred to a polyvinylidene difluoride membrane. Primary antibodies were diluted in Tris-buffered saline containing 0.05% Tween, 5% bovine serum albumin, and 0.02% sodium azide. Membranes were incubated overnight with primary antibodies at 4 °C. Dilutions for antibodies were as follows: VCL (Cell Signaling; catalog no.: 13901; clone E1E9V; dilution: 1:5000); CKB (Abcam; catalog no.: ab151579; dilution: 1:1000); and Anti-rabbit (Promega; catalog no.: W4011; dilution: 1:10,000). Results were visualized with chemiluminescence Western blotting substrates (Bio-Rad).

### RT–quantitative PCR

RNA samples (1–2 μg) were converted to complementary DNA (cDNA) using the High-Capacity cDNA Reverse Transcription Kit (Applied Biosystems; catalog no.: 4368814). The synthesized cDNA was subsequently analyzed through RT–quantitative PCR (qPCR). Each reaction contained 20 ng of cDNA along with 187.5 nmol of each primer, combined with GoTaq qPCR Master Mix (Promega; catalog no.: A6002). Reactions were performed in a 384-well plate format using the CFX384 Real-Time PCR System (Bio-Rad). Gene expression was normalized *via* the ΔΔCt method, with *TBP* mRNA serving as the reference gene. CFX Maestro 2017 was used for data collection. Primer sequences used for RT–qPCR can be found in the supporting information.

### CK activity

A coupled enzymatic reaction (pyruvate kinase and lactate dehydrogenase) was used to determine CK activity in the forward direction (*creatine + ATP* → *ADP + phosphocreatine*). Absorbance at 340 nm was measured to determine the NADH oxidation rate using a BioTek Synergy H1 plate reader in kinetic mode. Cells were lysed in buffer (50 mM Tris–HCl [pH 8.0], 150 mM NaCl, 1% IGEPAL [NP-40], 1 mM EDTA, and 20% glycerol). The assay was performed at 25 °C by supplementing assay buffer (20 mM MgCl_2_, 100 mM KCl, 5 μg oligomycin, and 50 mM Tris [pH 9.0]) with coupling substrates (5 mM ATP, 4 mM PEP, and 0.45 mM NADH), 40 μg of protein, and 10 mM creatine.

### Cell proliferation using IncuCyte

One thousand cells were seeded in a 96-well plate. Photos of the cell density were taken every 4 h for 5 days and analyzed by IncuCyte S3 live cell imaging.

### Wound healing assay

About 10 × 10^3^ cells were seeded in a 96-well plate to reach 100% confluency the next day. The scratch was made with IncuCyte WoundMaker. Cells were washed with 1X PBS, and the medium was refreshed. Scratch wound healing was analyzed over 24 h by IncuCyte S3 live cell imaging.

### Anoikis

Cells were treated with 5 μg/ml mitomycin for 4 h. Fifty thousand cells were seeded in a low-attachment plate (24-well format). Five days later, cells were counted manually using a hemocytometer after treatment with trypan blue dye.

### Cell migration

Cells were seeded in Transwell chambers (8 μm, 24-well format). DMEM (100 μl) containing 8000 cells was added to the upper chamber, and 600 μl of medium containing FBS as the chemoattractant (DMEM, 10% FBS, and 1% P–S) was added to the lower chamber. After overnight incubation, the nonmigrant cells were removed with a cotton swab, and the insert was immersed in 4% paraformaldehyde for 20 min at room temperature. Cells were stained with crystal violet. The bound crystal violet was eluted by adding 33% acetic acid and shaking for 10 min. The absorbance at 590 nm was measured using a plate reader.

### Animals

Immunodeficient NSG (NOD.Cg-*Prkdc*^*scid*^*Il2rg*^*tm1Wjl*^/SzJ) mice were purchased from Charles River Laboratories. Mouse experiments were performed according to procedures approved by the Animal Resource Centre at McGill University and complied with guidelines set by the Canadian Council of Animal Care.

### Tail vein injections

Tail vein injections were performed by injecting 1 × 10^6^ H1299 cells directly into the lateral tail vein of NSG males. After 5 weeks, all mice were sacrificed, lungs and livers were removed, and the metastatic lesion area was quantified. The metastatic burden in the lungs was quantified from two H&E-stained step sections. The metastatic burden in the livers was quantified from one H&E-stained section. The metastatic lesion area/organ area was quantified using the bioimage analysis software QuPath.

## Data availability

The data supporting the findings of this study are available within the article.

## Supporting information

This article contains supporting information.

## Conflict of interest

The authors declare that they have no conflicts of interest with the contents of this article.
