# Supplementary material for: Creatine kinase B promotes non–small cell lung cancer survival and metastasis
Source: J Biol Chem. 2025 Oct 8;301(11):110805. doi: 10.1016/j.jbc.2025.110805 (PMC12630352; doi:10.1016/j.jbc.2025.110805)
Supplement: Supplemental Figures [file mmc1.docx]

**SUPPLEMENTAL INFORMATION**

**Creatine Kinase B promotes non-small cell lung cancer survival and metastasis**

Mouna Tlili^1,3^, Bozena Samborska^1,3^, Charlotte Girondel^1,2^, Afnan Abu-Thuraia^1,2^, Qiaoqiao Zhang^1,3^, Jakub Bunk^1,3^, Mohammed F. Hussain^1,3^, Peter M. Siegel^1,2,3^ and Lawrence Kazak^1,3^*.

^1^Rosalind & Morris Goodman Cancer Institute, McGill University, Montreal, QC, H3A 1A3, Canada

^2^ Department of Medicine, McGill University, Montreal, QC, H3A 1A1, Canada

^3^Department of Biochemistry, McGill University, Montreal, QC, H3G 1Y6, Canada

*Correspondence to: [lawrence.kazak@mcgill.ca](mailto:lawrence.kazak@mcgill.ca)

**SUPPLEMENTAL FIGURE LEGENDS**

**
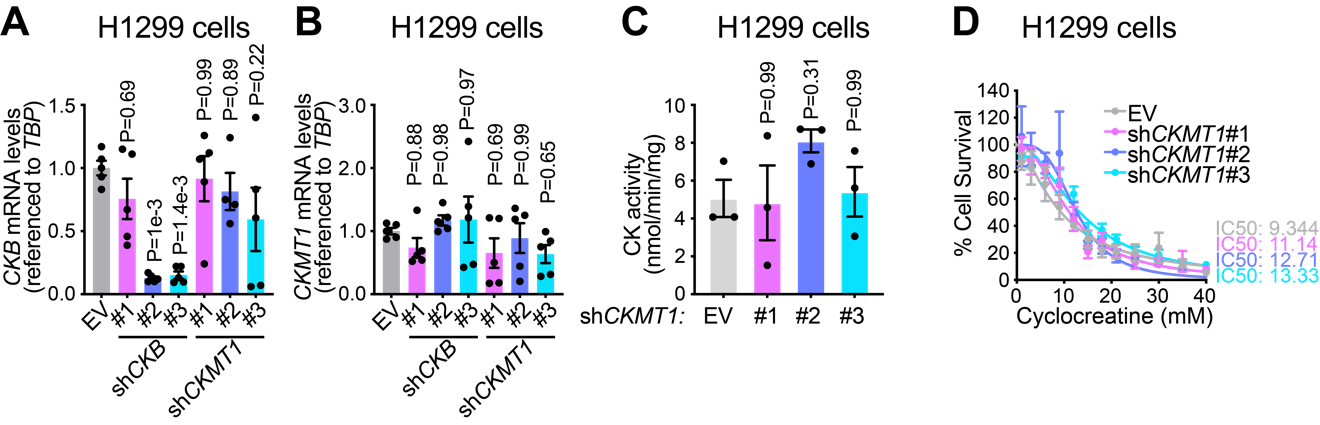
Fig. S1. CK activity in H1299 cells is not mediated by CKMT1.**

(A) Relative CKB mRNA expression in H1299 cells stably expressing empty vector (EV), shCKB#1, shCKB#2, shCKB#3, shCKMT1#1, shCKMT1#2, or shCKMT1#3 (n = 5, 5, 5, 5, 5, 4, 5).

(B) Relative CKMT1 mRNA expression in H1299 cells stably expressing empty vector (EV), shCKB#1, shCKB#2, shCKB#3, shCKMT1#1, shCKMT1#2, or shCKMT1#3 (n = 5 per group).

(C) CK activity in protein lysates from H1299 cells stably expressing EV, shCKMT1#1, shCKMT1#2, or shCKMT1#3 (n = 3 per group).

(D) Cell viability analysis, as determined by SRB staining, following three days of cyclocreatine treatment of H1299 cells stably expressing EV, shCKMT1#1, shCKMT1#2, or shCKMT1#3 (n = 6, 5, 4, 4).

Data are presented as mean ± s.e.m. n numbers are of biologically independent experiments. (A-C) One way ANOVA (Dunnett’s multiple comparison test).

**
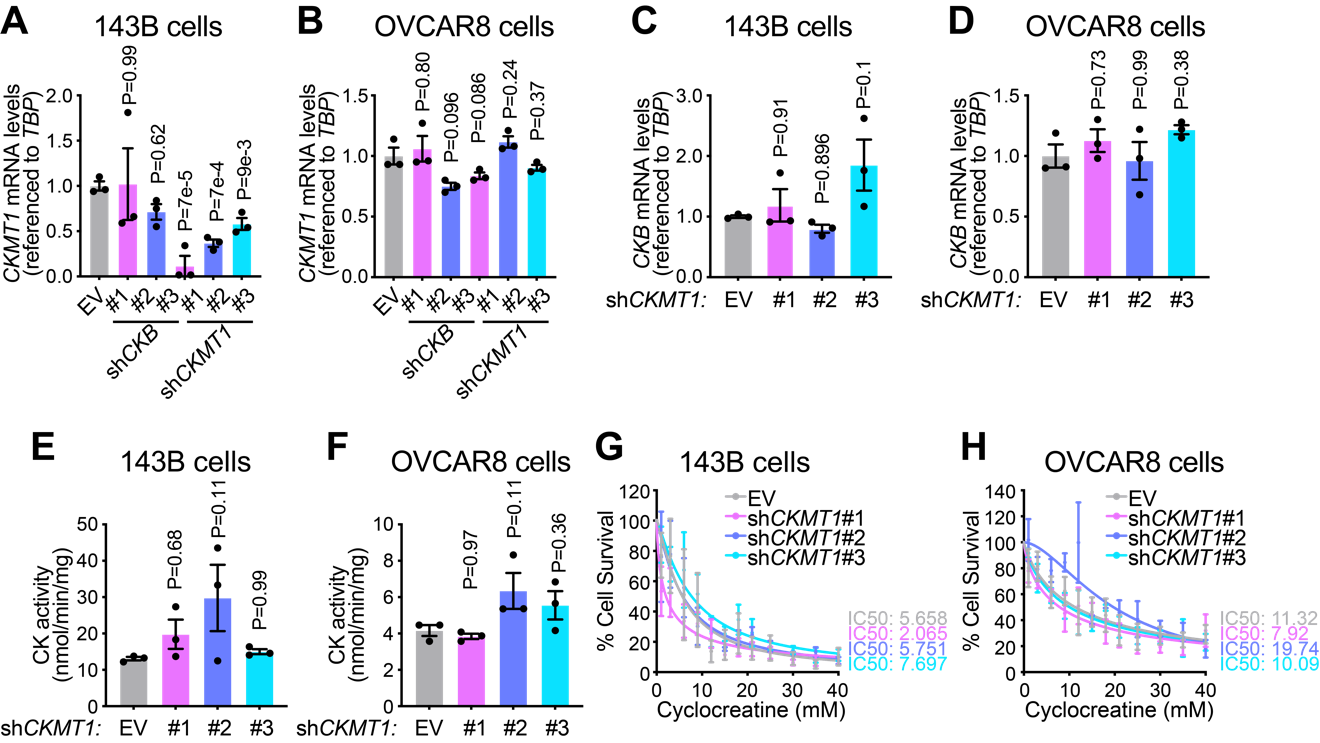
Fig. S2. CKMT1 is dispensable for CK activity in bone and ovarian cancer cells *in vitro*.**

(A-B) Relative CKMT1 mRNA expression in (A) 143B or (B) OVCAR8 cells stably expressing empty vector (EV), shCKB#1, shCKB#2, shCKB#3, shCKMT1#1, shCKMT1#2, or shCKMT1#3 (n = 3 per group).

(C-D) Relative CKB mRNA expression in (C) 143B or (D) OVCAR8 cells stably expressing EV, shCKMT1#1, shCKMT1#2, or shCKMT1#3 (n = 3 per group).

(E-F) CK activity in protein lysates from (E) 143B or (F) OVCAR8 cells stably expressing EV, shCKMT1#1, shCKMT1#2, or shCKMT1#3 (n = 3 per group).

(G) Cell viability analysis, as determined by SRB staining, following three days of cyclocreatine treatment of 143B cells stably expressing EV, shCKMT1#1, shCKMT1#2, or shCKMT1#3 (n = 4, 4, 4, 3).

(H) Cell viability analysis, as determined by SRB staining, following three days of cyclocreatine treatment of OVCAR8 cells stably expressing EV, shCKMT1#1, shCKMT1#2, or shCKMT1#3 (n = 4, 3, 4, 3).

Data are presented as mean ± s.e.m. n numbers are of biologically independent experiments. (A-F) One way ANOVA (Dunnett’s multiple comparison test).
